# Hyaluronic Acid (HA), Platelet-Rich Plasm and Extracorporeal Shock Wave Therapy (ESWT) promote human chondrocyte regeneration *in vitro* and ESWT-mediated increase of CD44 expression enhances their susceptibility to HA treatment

**DOI:** 10.1371/journal.pone.0218740

**Published:** 2019-06-28

**Authors:** Mario Vetrano, Danilo Ranieri, Monica Nanni, Antonio Pavan, Florence Malisan, Maria Chiara Vulpiani, Vincenzo Visco

**Affiliations:** 1 Department of Surgical and Medical Sciences and Translational Medicine, Faculty of Medicine and Psychology, "Sapienza" University of Rome, Rome, Italy; 2 Sant’Andrea University Hospital, Rome, Italy; 3 Department of Clinical and Molecular Medicine, Faculty of Medicine and Psychology, "Sapienza" University of Rome, Rome, Italy; 4 Department of Biomedicine and Prevention, University of Rome “Tor Vergata”, Rome, Italy; Mayo Clinic Minnesota, UNITED STATES

## Abstract

Novel strategies have been proposed for articular cartilage damage occurring during osteoarthritis (OA) and -among these- Extracorporeal Shock Wave Therapy (ESWT), intra-articular injections of Platelet-Rich Plasma (PRP) or Hyaluronic Acid (HA) revealed encouraging results. To investigate the possible mechanisms responsible for those clinical benefits, we established primary cultures of human chondrocytes derived from cartilage explants and measured the in vitro effects of ESW, PRP and HA therapies. After molecular/morphological cell characterization, we assessed those effects on the functional activities of the chondrocyte cell cultures, at the protein and molecular levels. ESWT significantly prevented the progressive dedifferentiation that spontaneously occurs during prolonged chondrocyte culture. We then attested the efficiency of all such treatments to stimulate the expression of markers of chondrogenic potential such as SOX9 and COL2A, to increase the Ki67 proliferation index as well as to antagonize the traditional marker of chondrosenescence p16INK4a (known as Cdkn2a). Furthermore, all our samples showed an ESW- and HA-mediated enhancement of migratory and anti-inflammatory activity onto the cytokine-rich environment characterizing OA. Taken together, those results suggest a regenerative effect of such therapies on primary human chondrocytes in vitro. Moreover, we also show for the first time that ESW treatment induces the surface expression of major hyaluronan cell receptor CD44 allowing the increase of COL2A/COL1A ratio upon HA administration. Therefore, this work suggests that ESW-induced CD44 overexpression enhances the in vitro cell susceptibility of human chondrocytes to HA, presumably favouring the repair of degenerated cartilage.

## Introduction

Osteoarthritis (OA) is considered the most common form of chronic arthritis involving different joints and characterized by progressive degeneration of cartilage[1, 2]. It is the result of an abnormal wound healing response, culminating in an imbalance between synthesis and degradation of extracellular matrix (ECM) provided by articular chondrocytes[3, 4]. In fact, it has been primarily documented that the presence of specific markers such as the chondrogenic master regulator SRY (sex-determining region Y) box 9 (SOX9) and Collagen type 2A (COL2A) characterizes healthy human cartilage, whereas in damaged OA cartilage there is a pathologic predominance of profibrotic Collagen type 1A (COL1A)[5, 6].

Unfortunately, human hyaline cartilage has a limited capacity for intrinsic regeneration and damage repair -probably due to poor vascularization- and it is now accepted that its maintenance depends on chondrocyte synthesis of several mediators[7, 8].

Nowadays, curative treatments for OA remain unsatisfactory, and its management focuses on the alleviation of symptoms, as recommended by the guidelines[9]. Current therapies are conservative, even though -for patients with severe OA- joint replacement can be required[10]. However, to reduce pain and induce a functional recovery, promising approaches using Extracorporeal Shock Wave Therapy (ESWT), Hyaluronic Acid (HA) or Platelets-Rich Plasma (PRP) were proposed[11–13].

Despite the shockwave efficacy in reducing symptoms, the molecular mechanisms evoked by such strategy are not yet completely understood, although the clinical benefits may be ascribed to induction of tissue regeneration[14–17].

HA is a major component of the cartilage ECM, and its molecular weight modification may lead to a compromised function of OA-affected joints[18]. For this reason, recent advances suggest that hyaluronan supplementation in OA patients, progressively affected by decreasing levels of HA concentration, may improve their clinical outcomes[19, 20].

Previous *in vitro* studies indicate that HA effects on cartilage tissue and cells are mediated through cluster differentiation 44 (CD44), the main hyaluronan receptor, whose expression in human chondrocytes can significantly influence the benefits of this therapy facilitating cell-matrix interaction[18, 21].

PRP is another promising treatment performed with autologous platelets purified from whole blood, whose advantages presumably depend on its anti-inflammatory and tissue healing properties through several mediators stored in the α–granules, which are supposed to improve tissue repair[13, 22].

Notwithstanding several difficulties to perform cultures of human chondrocytes -also due to the paucity of cells within articular cartilage-, tissue engineering and cell-based therapy are nowadays explored[1, 2]. However, their main disadvantage concerns the chondrocyte tendency to progressively dedifferentiate in vitro, which is not suitable for cartilage regeneration and also increases the risk of developing fibrosis[6, 7, 23].

Furthermore, one of the major limitations in healing the damaged cartilage is represented by cellular senescence associated with OA and characterized by an advanced age (or trauma-dependent) decline of tissue regeneration capacity[1, 2, 4, 7, 24, 25]. This chondrosenescence is intimately linked to several factors and may be measured in primary cultured human chondrocytes by different biomarkers of cell senescence, such as the p16^INK4a^ protein expression (also known as Cdkn2a)[26–28]. It is now widely believed that novel strategies should be explored to counteract chondrocyte dedifferentiation and senescence *in vitro*.

Many studies suggest that a further contribution to OA onset and progression is represented by an inflammatory environment characterizing either early or advanced disease, such as an increase in proinflammatory catabolic (e.g., TNF-α, IL-6 and IL-17A) and a decrease in anti-inflammatory regenerative cytokines (e.g. IL-10) can be considered an etiologic factor playing important roles also in the development and evolution of the disease[8, 18, 29–31].

In the present work, we established primary cultures of human chondrocytes explanted from femoral head articular cartilage of six patients undergoing total hip replacement, to evaluate the effects of their exposure to ESWT, HA and PRP, and to achieve a better understanding of the molecular mechanisms triggered *in vivo* by such treatments.

Our results indicate that these therapies promote a regenerative effect on primary human chondrocytes in vitro.

## Material and methods

### Tissue samples, human primary cultured chondrocytes and treatments

The IRB (Institutional Review Board) of ‘‘Sapienza'' University and Sant’ Andrea Hospital (Rome, Italy) approved this study, and each patient gave their written informed consent to the experimental research, following the principles outlined in the Declaration of Helsinki.

Four primary cultures of human chondrocytes have been established using femoral head articular cartilage biopsies obtained from six patients enrolled in this study (aged 58–67 years), who underwent total hip replacement for a prolonged history of OA. Tissue biopsies were derived from femoral head articular cartilage, cut into small pieces (2.5–3.0 mm^3^) and then digested with 2 mg/ml collagenase type I (Gibco, USA) for 1 hour at 37°C. The samples were centrifuged (1000 rpm for 10'), the supernatants discarded, and the pellet was cultured in a sterile flask (Falcon, BD, USA), in Ham’s F12 medium, supplemented with 10% fetal bovine serum (FBS, Hyclone, Euroclone, Italy) and 1% penicillin/streptomycin/glutamine solution (Gibco, USA). Primary cultures were expanded and, once they reached 80% confluence, at passage three (P3), they were used for the following experiments. Chondrocytes were detached with 1 ml of 0.05% trypsin/ethylenediaminetetraacetic acid (EDTA, Euroclone, Italy), counted and then used for all experiments.

For ESWT, primary cultured chondrocytes were treated with the shockwave generator at a dose of 0.14 mJ/mm^2^ energy level and 1000 impulses, as previously described[14, 17, 32], whereas the control group was maintained in conventional medium, without previous shockwave exposure. Intensity and energy levels were previously selected[14, 17, 32] to maximize the therapeutic effects without significantly decrease the cell viability. The shockwave treatment was applied by an electromagnetic shockwave generator MODULITH SLK (STORZ MEDICAL AG, Switzerland). A cryogenic vial (Corning Incorporated, NY, USA) containing 10^6^ cells/ml was placed on the generator with a coupling gel (Aquasonic 100; Parker Laboratories, USA) to minimize the loss of energy at the interface between the head of the device and the cryovial. The tube was placed precisely in the focus of the application pad under ultrasonographic control. Then, cells were cultured in a 6 cm dish for 72 hours.

For HA experiments, we used hyaluronic acid hybrid complex commercialized by IBSA Farmaceutici (Italy), (ratio 1:1) with H-HA (MW: 1200 ±100 kDa) and L-HA (MW: 100 ± 10 kDa) prepared as reported[24]. HA stock solution was mixed with culture media and the final concentration of hyaluronan mixture was 0.5%. Then, 10^6^ chondrocytes were seeded in a 6 cm dish and cultured overnight before treatment. Finally, cells were treated with the HA solution for 72 hours.

For ESWT pretreatment followed by HA exposure, after shockwave cells were cultured for an additional 48 hours in the HA-containing medium.

Platelet-rich plasma (PRP) was supplied by Immunohematology and Transfusion Unit (SIMT) of the Sant’Andrea Hospital. 20 ml of blood were collected using two tubes of RegenKit BCT-3 (Regenlab, Le Mont-sur-Lausanne, Switzerland) for each donor. All donors gave their informed consent. The tubes were then centrifuged for 12 min (1300 to 1500 rpm) resulting in 6–7 ml of PRP. To obtain a higher cellular concentration, we delicately removed 2 ml of the upper layer of the platelet-poor supernatant (PPP) and the remaining cellular deposit was collected in one tube (mean platelets concentration of 0.9–1.1 × 10^9^ ml). To induce PRP to release growth factors and cytokines, we added five NIH units of thrombin followed by calcium gluconate 1:20, allowing PRP to clot for 5 minutes at 37°C. The clot was centrifuged for 10 minutes at 600 rpm to obtain supernatants rich in growth factors and cytokines released from activated platelets[33]. Tubes were centrifuged at 600 rpm for 6 min, and the plasma layer was collected avoiding aspiration of the buffy coat, the resulting product includes fibrinogen. Platelets and leukocytes in PRP were counted by a Coulter Counter (Beckman, Brea, CA, USA). 10^6^ chondrocytes were seeded in a 6 cm dish and cultured overnight and then treated with such preparation for 72 hours. For all our treatments, 10^6^ chondrocytes were seeded in a 6 cm dish and cultured for 72 hours as control.

### Morphological evaluation

Chondrocytes were seeded (10^6^ cells/dish), grown at 37°C for 12 days and then assessed for cellular morphology. At day 4, 8, and 12 photomicrographs were recorded using an Axiovert 200 inverted microscope (Carl Zeiss, Micro lmaging, GmbH, Germany), equipped with a Camera Power Shot A640 (Canon, Japan). According to previous studies on the differentiation of human skin fibroblasts[34], chondrocytes were distinct for their phenotype into two subpopulations: Elongated (E) and Ovoid (O) cells[17]. A cell count of ten photomicrographs performed quantitative analysis for each time point. Results are shown as means ± SD. Student’s *t-test* was performed, and significance levels have been defined as p<0.05.

### Scratch assay

Cells treated as above described were seeded on 6 cm dishes and incubated for 24 hours at 37°C. Untreated cells were used as control. Then a standardized cell-free area was introduced by scraping the monolayer with a sterile tip, as previously described[35]. After intensive wash, the remaining cells were incubated for further 48 hours, treated as above, then fixed with 4% paraformaldehyde for 30 min at 25°C; photographs were taken using an Axiovert 200 inverted microscope (Zeiss, Germany). Some plates were fixed and photographed immediately after scratching representing a T0 control. Migration was quantitated by a measure of the recovered scratch area, performed using the Axiovision software (Zeiss, Germany). The data now presented is a mean percentage ± SD of residual open area compared to the respective cell-free surface in T0. Results are shown as means ± SD. Student’s *t-test* was performed, and significance levels have been defined as p<0.05.

### Immunofluorescence

Chondrocytes primary cultures, grown on coverslips and treated as above described, were fixed with 4% paraformaldehyde followed by treatment with glycine 0.1 M for 20 min at 25°C and with 0.1% Triton X-100 for additional five min. at 25°C to allow permeabilization. Cells were then incubated, alternatively, with the following primary antibodies: the mouse monoclonal anti-p16 (1:100 in PBS; BIO-p16, UCS Diagnostic, Italy) and the rabbit polyclonal antibody anti-Ki67 (1:50; Zymed Laboratories, USA) and the directly conjugated mouse monoclonal anti-CD44-FITC (Becton and Dickinson Company, USA). The primary antibodies were visualized, after washing with PBS, using the goat anti-mouse IgG-Alexa Fluor 488 (1:200 in PBS; Life Technologies, USA) and the goat anti-rabbit IgG-FITC (1:200; Jackson Immunoresearch Laboratories, USA), for 30 min. at 25°C. Nuclei were stained with 4’-6’-diamido-2-phenylindole dihydrochloride (DAPI) (1:10000 in PBS, Sigma Chemical Company, USA). Finally, coverslips were mounted with Mowiol in PBS for observation. Fluorescence signals were analyzed by conventional fluorescence with an ApoTome System (Zeiss, Germany) connected with an Axiovert 200 microscope (Zeiss); image analysis was then performed by a software Axiovision (Zeiss, Germany). Quantitative analysis of the fluorescence intensity was performed by the Axiovision software (Zeiss, Germany), analyzing 10 different fields randomly taken from three independent experiments as reported in other studies[36]. Results are shown as means ± SD. Student’s *t-test* was performed, and significance levels have been defined as p<0.05.

### Cytokine levels in supernatants of human primary cultured chondrocytes

In cells treated as above, cytokine supernatant levels [IL-6, IL-17A, IL-10, and tumour necrosis factor (TNF-α] were measured simultaneously by multiple immunoassay kits (Human Magnetic Luminex Assay, R&D System Inc. Bio-Techne brand) following the manufacturer’s instructions and using the Luminex Technology. Quantitative data were obtained by the Luminex-200 MagPix system (Luminex Corporation, TX, USA), and the data were analyzed using Luminex 200 software. Quantitative analysis of cytokine secretion was performed as mean fluorescence intensity (MFI) as above. Results are shown as means ± SD. Student’s *t-test* was performed, and significance levels have been defined as p<0.05.

### Primers

Oligonucleotide primers necessary for target genes and the housekeeping gene were chosen utilizing the online tool Primer-BLAST and purchased from Invitrogen (USA).The following primers were used: for the transcription factor, SOX9 target gene: 5’-CCCCCAACGCCATCTTCAA-3’ (sense), 5’-CTGGGATTGCCCCGAGTG-3’ (anti- sense); for the type II collagen, COL2A target gene: 5’-ACACTGGGACTGTCCTCTGCGA-3’ (sense), 5’-CCTTTGGTCCTGGTTGCCCACTG-3’ (anti-sense); for the type I collagen, COL1A target gene: 5’-ACATGTTCAGCTTTGTGGACCTCCG-3’ (sense), 5’-ACGCAGGTGATTGGTGGGATGTCT-3’ (anti-sense); for the cyclin-dependent kinase Inhibitor 2A CDKN2A/p16 target gene: 5’-CGTGGACCTGGCTGAGGA-3’ (sense), 5’- AATCGGGGATGTCTGAGGGA-3’ (anti-sense); for CD44 target gene: 5’-TCCAACACCTCCCAGTATGACA-3’ (sense), 5’-GGCAGGTCTGTGACTGATGTACA-3’ (anti-sense); and for the 18S rRNA housekeeping gene: 5’-AACCAACCCGGTCAGCCCCT-3’ (sense), 5’-TTCGAATGGGTCGTCGCCGC-3’ (antisense). For each primer pair, we performed no-template control and no-reverse-transcriptase control assays, which produced weak signals.

### RNA extraction and cDNA synthesis

RNA was extracted using the TRIzol (Invitrogen) according to manufacturer’s instructions and eluted with 0.1% diethylpyrocarbonate (DEPC)-treated water. Each sample was treated with DNAase I (Invitrogen, USA). Total RNA concentration was quantitated by spectrophotometry; 1 μg of total RNA was used to reverse transcription using iScriptTM cDNA synthesis kit (Bio-Rad, USA) according to manufacturer’s instructions.

### PCR amplification and real-time quantitation

Real-time RT-PCR was performed using the iCycler Real-Time Detection System (iQ5 Bio-Rad, USA) with optimized PCR conditions. The reaction was carried out in 96-well plate using iQ SYBR Green Supermix (Bio-Rad, USA) adding forward and reverse primers for each gene and one μl of diluted template cDNA to a final reaction volume of 15 μl. All assays included a negative control and were replicated three times. The thermal cycling program was performed as described[33]. Real-time quantitation was performed with the help of the iCycler IQ optical system software version 3.0a (Bio-Rad, USA), according to the manufacturer’s manual. Results are reported as mean ± SD from three different experiments in triplicate. Student’s *t-test* was performed, and significance levels have been defined as p<0.05.

## Results

### Characterization of primary cultured human chondrocytes

Primary cultured human chondrocytes have been used to evaluate the possible mechanisms responsible for the clinical benefits encountered after promising strategies as Extracorporeal Shock Wave Therapy (ESWT), Hyaluronic Acid (HA) and Platelets-Rich Plasma (PRP) for the management of several musculoskeletal disorders.

We established four primary cultures of human chondrocytes derived from femoral head articular cartilage of six patients (aged 58–67 years) undergoing total hip replacement for a prolonged history of OA.

**Fig 1:**
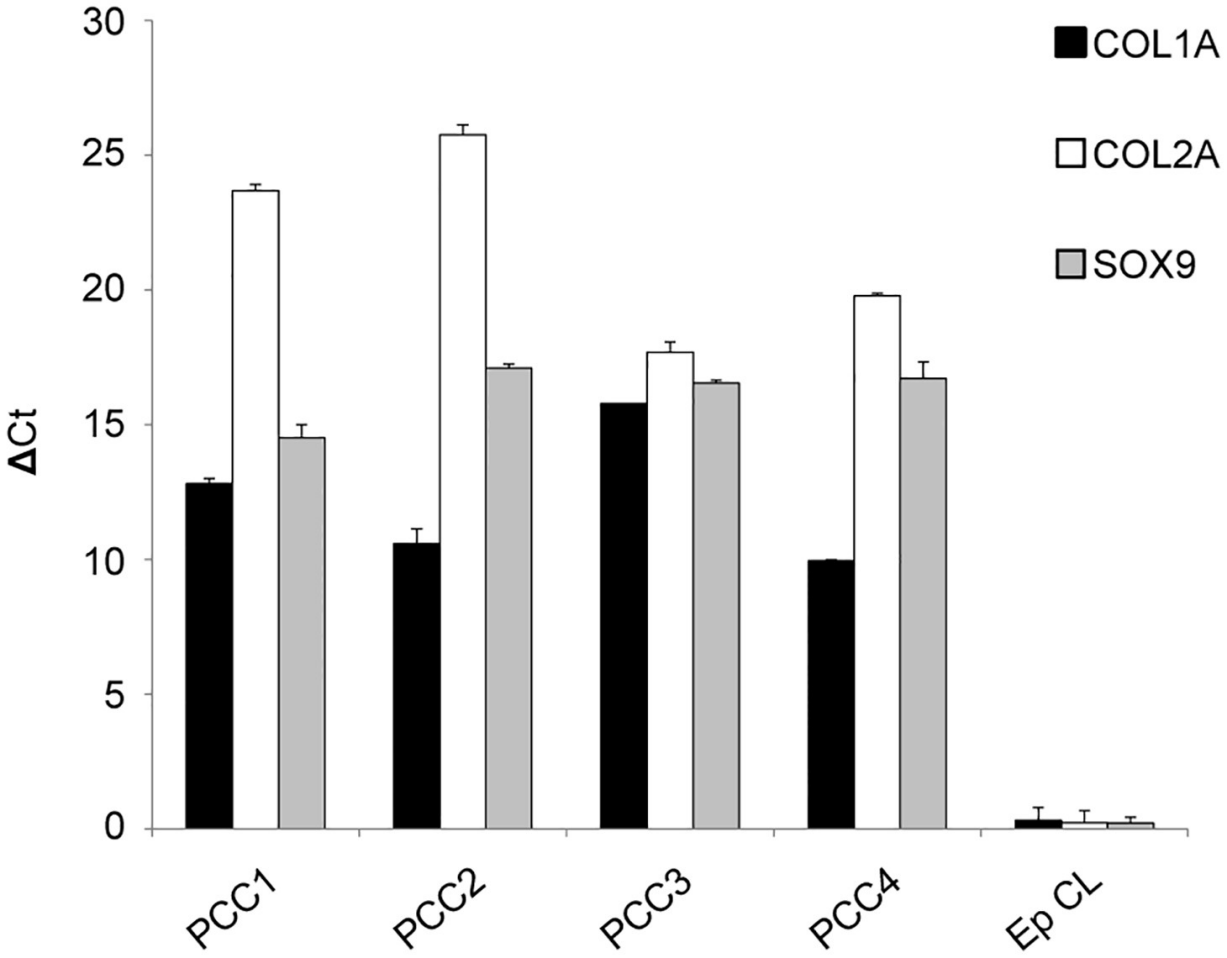
Characterization of the four primary cultures of chondrocytes derived from femoral head articular cartilage biopsies. Molecular analysis of the expression of typical chondrocyte markers: SOX9, COL1A and COL2A. Real-time RT PCR quantitated mRNA transcript levels. Results were obtained from four different primary cultures of chondrocytes.

First, conventional chondrocyte status markers -including SOX9, COL1A and COL2A- have been evaluated by RT-PCR. The molecular analysis permitted to detect the simultaneous expression of all gene markers in the samples, assessing the quality of our cell cultures (Fig 1).

**Fig 2:**
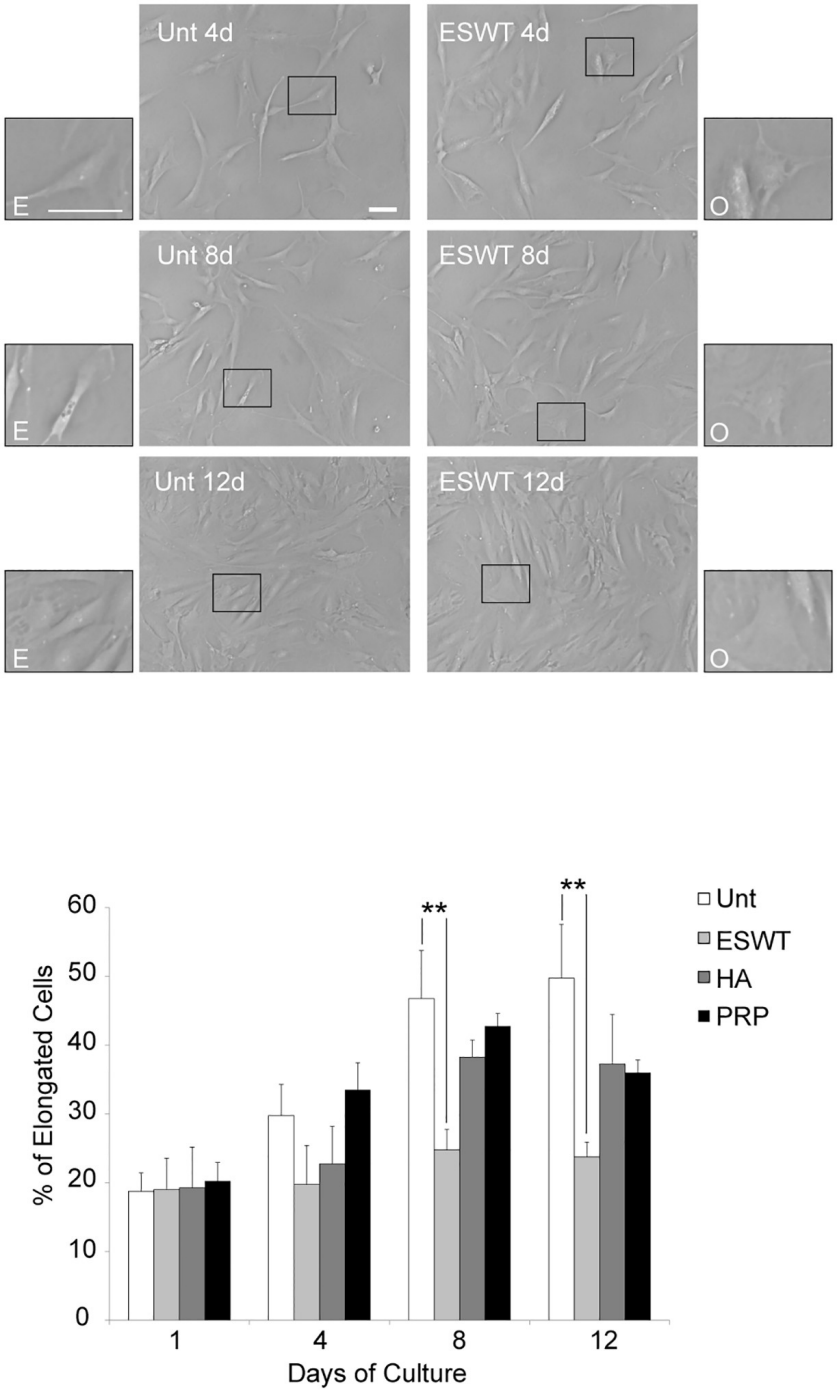
ESWT significantly antagonized the *in vitro* phenotypic dedifferentiation of primary human chondrocytes. Quantitative analysis of cell morphology performed over a 12-day (d) period in human primary cultured cells was derived from 4 different explants, then exposed to ESW, HA and PRP or left untreated as control (Unt). According to morphological criteria, cells defined as O (ovoid) or E (elongated) were counted from five different fields taken randomly. Results were performed by assessing the percentage of elongated cells and are expressed as mean values ±SD. Student’s *t-test* was calculated to evaluate significant differences: values of *p*<0.001 (**) were considered statistically significant vs the corresponding untreated. Photomicrographs are representative of one single culture. Bar 50 μm.

In this model, the human chondrocytes seeded in vitro showed different patterns of cell morphology. Indeed, inside the cultures, a first and prevalent group of classically rounded, polygonal, ovoid (O) and enlarged chondrocytes, as well as a second group of elongated (E), flattened, fibroblast-like cells were observed (Fig 2).

### ESWT significantly antagonized the *in vitro* phenotypic dedifferentiation of primary human chondrocytes

We compared untreated long-term cell cultures with cultures exposed to either ESWT, or HA or PRP.

A quantitative evaluation to check the percentages of E and O morphologies in four different cultures was performed, showing a significant correlation between the cellular shapes and the various treatments. In particular, the analysis conducted after one day up to the following 12 days (as documented in Fig 2 by photomicrographs of representative fields) showed phenotypic dedifferentiation observable in control cells, consisting in a significant increase of E vs O phenotype. Interestingly, only the shockwave exposure prevented this morphological modification after eight up to 12 days, whereas no significant differences have been detected in PRP- and HA-treated cells: ESWT significantly antagonized this progressive drift from E to O morphology (Fig 2).

### The expression of SOX9 is increased and the functional shift COL2A towards COL1A is impaired in human primary cultured chondrocytes in response to ESWT, HA and PRP

Thus, we compared the molecular expression of collagen type IA (COL1A: fibrotic marker whose expression is enhanced during chondrocyte dedifferentiation) and type IIA (COL2A: a typical chondrogenic marker of the functionally active chondrocyte), as well as the expression of SOX9 (the chondrogenic master regulator).

**Fig 3:**
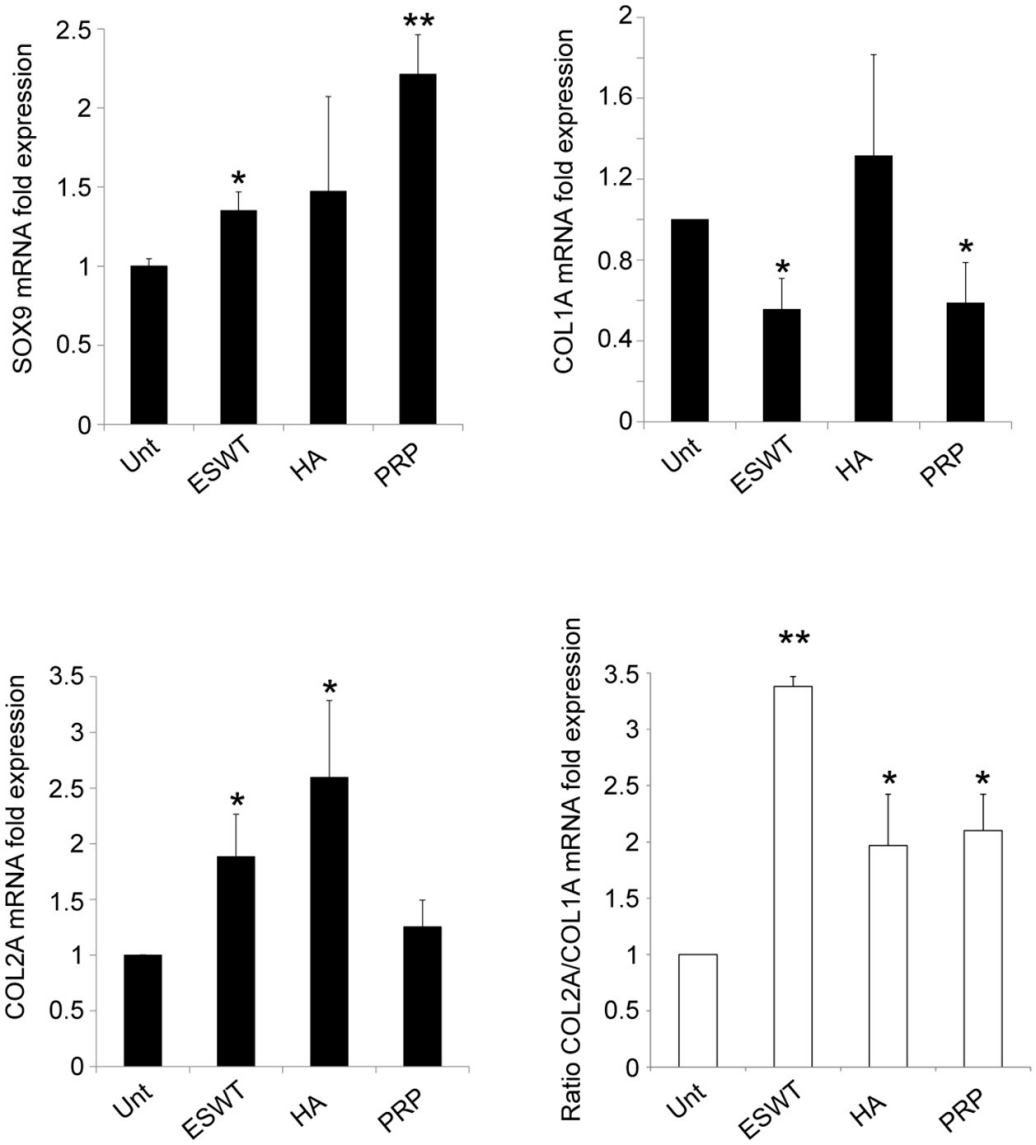
The expression of SOX9 is increased and the functional shift COL2A towards COL1A is impaired in human primary cultured chondrocytes in response to ESWT, HA and PRP. Real-time RT PCR quantitated mRNA transcript levels. The expression of SOX9, COL1A, COL2A and COL1A/COL1A ratio was calculated. Results reported in the graph were obtained from four different cultures and represent the mean values ±SD obtained from three independent experiments. Student’s *t-test* was calculated to evaluate significant differences: values *p*<0.001 (**) and *p*<0.05 (*) were considered statistically significant vs the corresponding untreated.

Our treatments (more significantly PRP and ESWT) impair the functional shift of COL2A- toward COL1A-expressing cultured chondrocytes and upregulate the levels of SOX9. To confirm the preservation of chondrocyte phenotype, the COL2A/COL1A ratio was also calculated (Fig 3).

### Proliferation and repair is stimulated by ESWT, HA and PRP treatment in chondrocyte cultures

**Fig 4:**
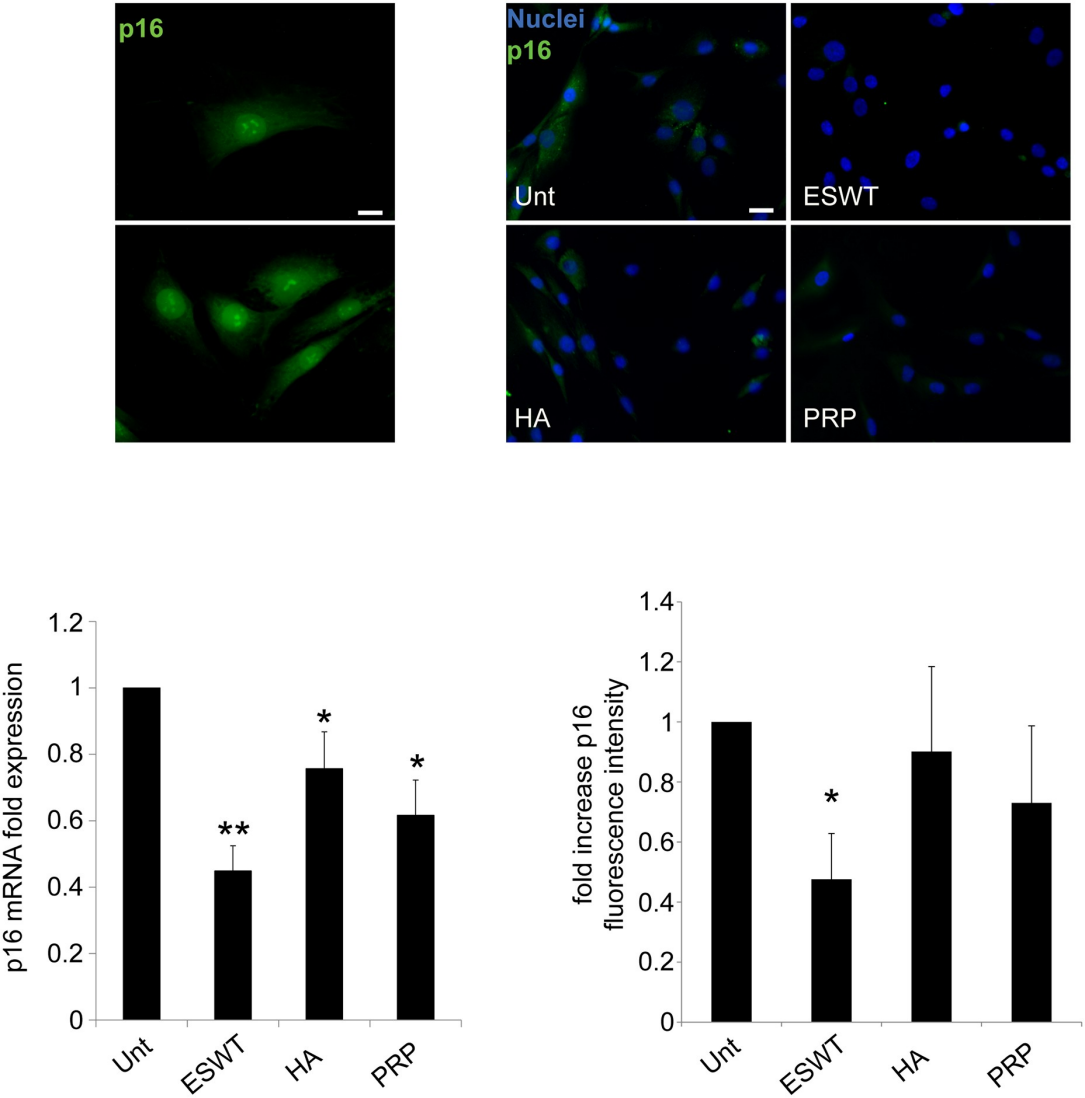
p16^INK4a^ expression is reduced in chondrocyte cultures in response to ESWT, HA and PRP. Immunofluorescence analysis of p16^INK4a^ expression was achieved with anti-p16 immunolabeling (green). Nuclei are stained with DAPI. Photomicrographs are representative of one single culture. Bar 20 μm. Photomicrographs show a typical nuclear and cytoplasmic immunolabeling of p16 ^INK4a^ positive cells. Quantitative immunofluorescence analysis of p16 was performed as described in material and methods. p16 mRNA transcript levels after ESWT, HA and PRP, were quantitated by real-time RT PCR. Results reported in the graph were obtained from four different cultures and represent the mean values ±SD obtained from three independent experiments. Student’s *t-test* was calculated to evaluate significant differences: values as *p*<0.001 (**) and *p*<0.05 (*) were considered statistically significant vs the corresponding untreated.

**Fig 5:**
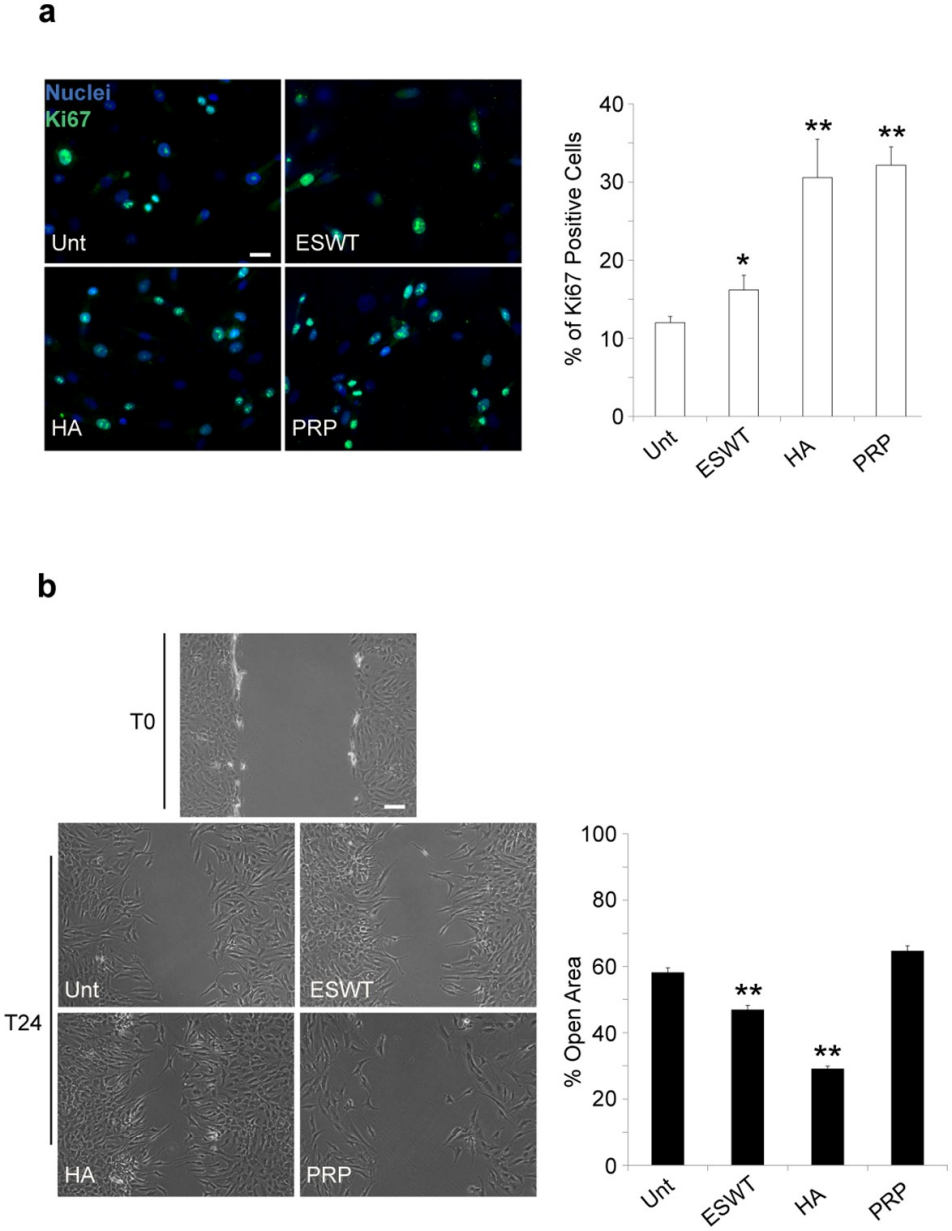
Proliferation and repair is stimulated by ESWT, HA and PRP treatment in chondrocyte cultures. Analysis of proliferation and migration induced by ESWT, HA and PRP were performed as described in material and methods. (a) *ESWT*, *HA and PRP effects on cell proliferation of chondrocyte cultures*. Immunolabeling with anti-Ki67 antibody (green) was achieved on cell cultures after ESW, HA and PRP exposure for 24 days (T24). Nuclei are stained with DAPI. Photomicrographs are representative of one single culture. Bar 20 μm.Quantitative immunofluorescence analysis of proliferation induced by ESWT, HA and PRP was performed as described in material and methods. Results are shown as means ±SD. Student’s *t-test* was performed, and significance levels have been defined as *p*<0.001 (**) and *p*<0.05 (*). (b) *ESWT*, *HA and PRP effects on cell migration of chondrocyte cultures*. Results reported in the graph were obtained from four different cultures and represent the mean percentage ±SD of a residual open area as described in material and methods. Results are shown as means percentage ±SD. Student’s *t-test* was performed, and significance levels have been defined as p<0.001 (**) and p<0.05 (*). Bar 50 μm.

We then analyzed (both at the protein and molecular level) the expression of p16^INK4a^ (also known as Cdkn2a), a specific biomarker of cell ageing. Our data support a possible role of shockwave (mainly), PRP and HA in retarding chondrosenescence, as indicated by the significant decrease of p16^INK4a^ expression in treated, compared with untreated, chondrocytes. Those results were confirmed at protein level by quantitative immunofluorescence evaluation and at the molecular level by mRNA analysis (Fig 4). Following these data, ESW- HA- and PRP-treated cells, after 6-well plates seeding, were also analyzed in immunofluorescence for the expression of Ki67, a nuclear marker of cycling cells. Quantitative analysis -measured by the percentage of Ki67^+^ cells- revealed a proliferative stimulus of such treatments on our cultures, mainly after three days. Those effects persisted after five days exclusively for PRP and HA exposure, becoming not significant in ESW-treated chondrocytes (Fig 5). Using the algorithm [http://www.doubling-time.com/] provided by Widera et al.[37], untreated cells compared with PRP and HA-treated cells showed at day three a threefold increase in doubling time, although this rate resulted significantly diminished at day five (Fig 5A). At the same time, migratory behavior was assessed by a scratch test, able to mimic a possible in vitro-induced cartilage repair. Cell migration was quantified measuring the mean of recovered areas after scratching (see Material and Methods paragraph). The HA- and ESW-exposure promoted a significant decrease in residual open field and a typical migratory phenotype in most of the chondrocytes, while no considerable outcome was observed after PRP-treatment. The quantitative analysis demonstrates that the HA- and ESWT-mediated motility was significantly higher compared to untreated control (p<0.01) (Fig 5B).

### Pro-inflammatory cytokine secretion is reduced whereas anti-inflammatory IL-10 level is increased in supernatants collected from chondrocyte cultures treated with ESWT and HA

**Fig 6:**
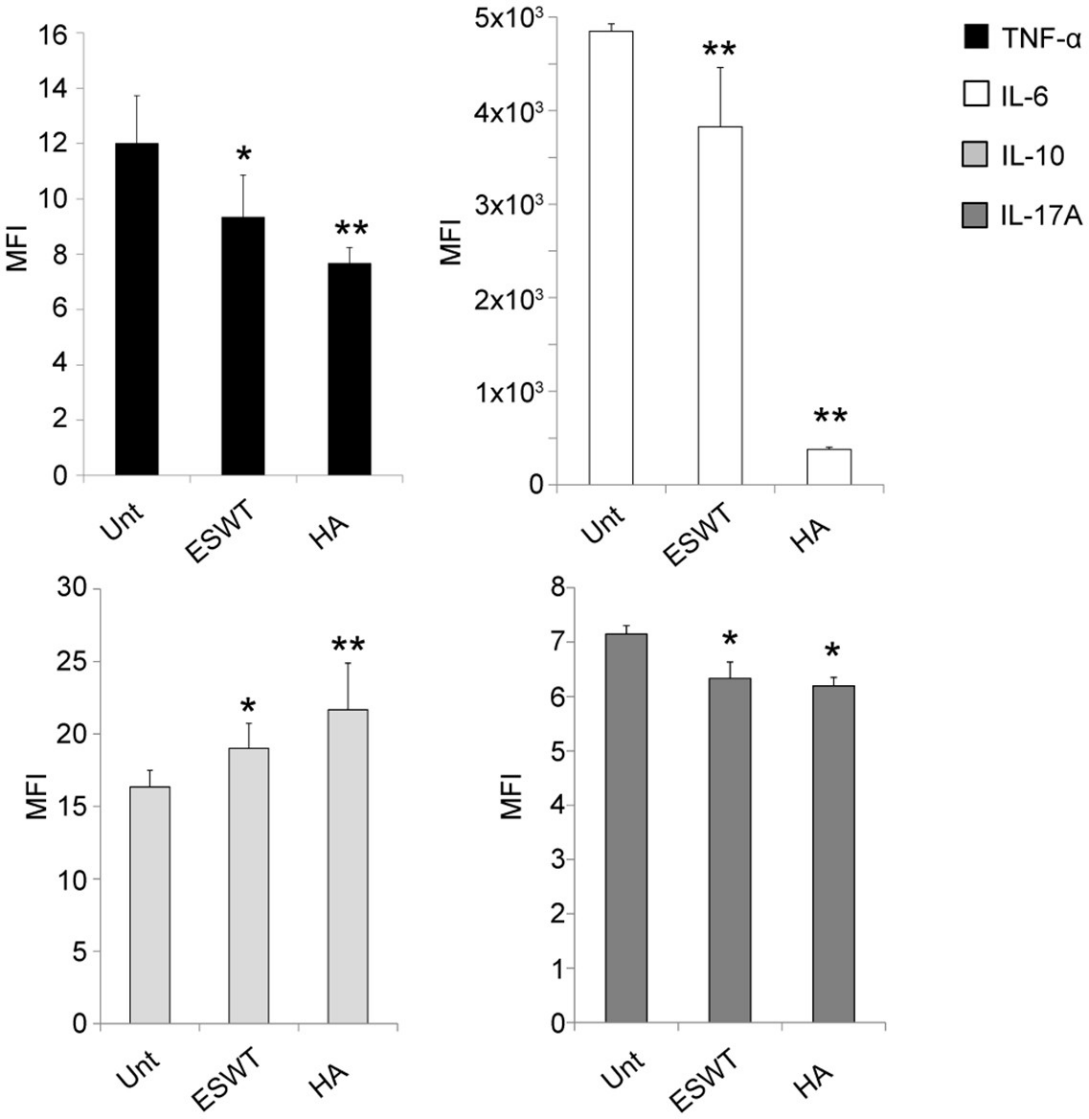
Proinflammatory Cytokine secretion is reduced whereas anti-inflammatory IL-10 level is increased in supernatants collected from chondrocyte cultures treated with ESWT and HA. Results reported in the graph represent the means fluorescence intensity (MFI) ±SD of 4 different cultures, as described above. Student’s *t-test* was performed, and significance levels have been defined as p<0.001 (**) and p<0.05 (*).

We measured the secretion of inflammation-related cytokines (TNF alpha, IL-6, IL-10 and IL-17A) in supernatants of cultured chondrocytes, collected 5 days after ESW and HA exposure (Fig 6). The cytokine secretion was not evaluated in PRP-treated cells, because it would not have been possible to understand if these were produced by chondrocytes or rather directly released by platelet degranulation. Interestingly, HA and ESWT exposure significantly inhibited IL-6, TNF-α and IL-17A, while stimulated IL-10 production (Fig 6). The analysis of cytokines showed decreased values for pro-inflammatory TNF-α, IL-6 and IL-17A and increased for anti-inflammatory IL-10.

### Functional effect of CD44 up-regulation mediated by ESWT

**Fig 7:**
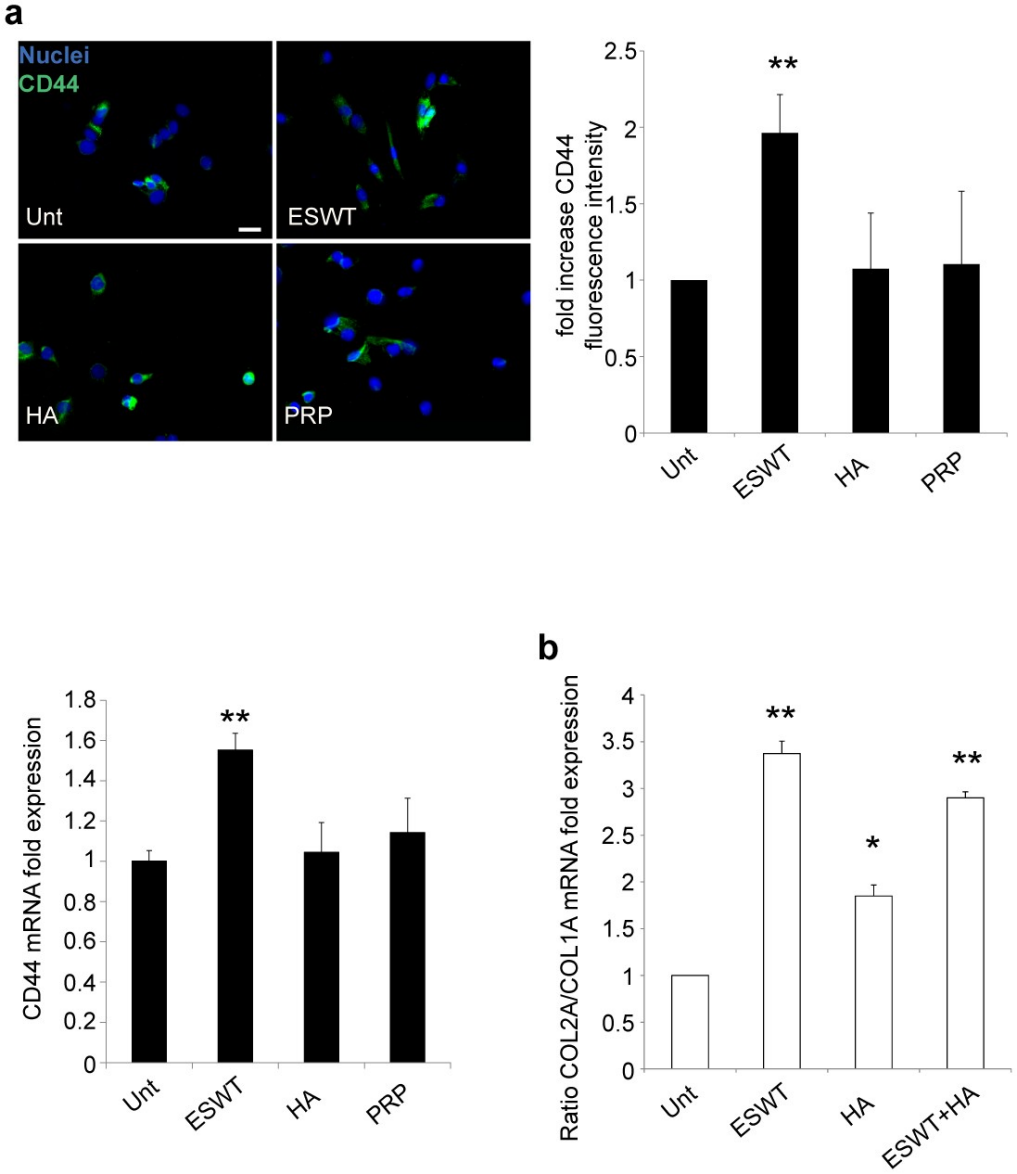
ESWT enhances CD44 expression and increases COL2A expression in chondrocyte cultures upon HA treatment. (a) Immunofluorescence analysis of CD44 expression induced by ESWT, HA and PRP was performed as described above. Immunolabeling with anti-CD44 (green) was achieved on cell cultures after ESW, HA and PRP exposure. Nuclei are stained with DAPI. Photomicrographs are representative of one single culture. Bar 20 μm. CD44 mRNA transcript levels after ESWT, HA and PRP were quantitated by real-time RT PCR. Results reported in the graph were obtained from four different cultures and represent the mean values ±SE obtained from three independent experiments. Student’s *t-test* was calculated to evaluate significant differences: values as *p*<0.001 (**) and *p*<0.05 (*) were considered statistically significant vs the corresponding untreated. (b) ESWT, HA, and ESWT pretreatment followed by HA (ESWT+HA) effects on the ratio of COL2A/COL1A expression. COL2A/COL1A ratio mRNA transcript levels after ESWT, HA, and ESWT+HA were quantitated by real-time RT PCR. Results reported in the graph were obtained from four different cultures and represent the mean values ±SE obtained from three independent experiments. Student’s *t-test* was calculated to evaluate significant differences: values as p<0.001 (**) and p<0.05 (*) were considered statistically significant vs the corresponding untreated.

Considering the described in vitro beneficial effects of HA, we quantified the surface expression of the major Hyaluronan cell receptor -CD44- on primary cultured human chondrocytes. The ubiquitous expression of CD44 in all human cultured chondrocytes was analyzed by Immunofluorescence microscopy. After only 3 days following ESW exposure, CD44 was significantly enhanced, whereas no relevant differences after HA- and PRP-treatments were observed (Fig 7). This result was first obtained by immunofluorescence and expressed in a quantitative analysis of the signal as fold increase CD44 fluorescence intensity (Fig 7A). Such an increase in CD44 cell surface protein expression was then confirmed at the molecular level by RT-PCR. Again, 3 days after ESWT, mRNA fold expression of CD44 was significantly enhanced compared to untreated control cells (Fig 7A).

Intriguingly, we also measured the functional effect of CD44 overexpression on the activity of HA treatment: in ESW-stimulated cells, HMW-HA added in the culture medium significantly increases the chondrogenic COL2A expression, as documented by the enhanced COL2A/COL1A ratio shown in Fig 7B.

## Discussion

In this study, we observe in vitro that HA, PRP and ESWT promote regeneration and attenuate human chondrocyte degeneration of osteoarthritic cells explanted by patients subjected to total hip replacement. In addition, we show for the first time that ESWT-mediated increase of CD44 expression enhances their susceptibility to HA treatment.

Currently, novel treatments (including HA, PRP and ESWT) of patients with severe OA are administered to alleviate the symptoms to defer joint replacement surgery and to enhance the poor regenerative potential of human cartilage[38]. Nevertheless, there is an ongoing debate over the exact mechanisms underlying the effectiveness of such therapies. In order to develop more efficient protocols, a better comprehension of the underlying mechanisms is required. This research aimed to investigate the *in vitro* biological effects of non-surgical treatments for OA on osteoarthritic chondrocytes explanted by patients subjected to total hip arthroplasty.

Each successful cell-based innovative therapy for cartilage repair showed two main limitations: 1) the naturally occurring cell dedifferentiation during long-term *in vitro* expansion[6, 7, 23, 25, 39] and 2) the chondrocyte senescence, probably triggered by different inflammatory stimuli regulating the balance of anabolic/catabolic activities in OA[26–28].

In the current work, we emphasized that -although with different modalities- ESWT, HA and PRP can help manage those restrictions affecting the cartilage repair capacity, confirming a possible relationship in the pathways involved in the dedifferentiation and senescence of human chondrocytes[4, 26].

First, we observed that ESWT significantly prevents the phenotypic shift of normal chondrocytes toward fibroblast-like cell dedifferentiation spontaneously occurring during prolonged cell culture, as already confirmed by previous studies[6, 7, 23, 25]. This is further confirmed by its capacity to impair such functional shift in expressing the fibrotic COL1A instead of chondrogenic COL2A marker, previously ascertained during dedifferentiation by several authors[6, 7, 23]. Then, we evidenced that ESWT and PRP exposure not only strongly decreases the expression of the senescence marker p16/CDKN2A, but also upregulates the levels of the chondrogenic markers SOX9 and COL2A.

Furthermore, after ESWT, HA and PRP stimulations, cultured chondrocytes enhance proliferation and migration, which represent essential prerequisites for tissue repair[39, 40].

OA is characterized by different stages of inflammation, playing a crucial role in cartilage remodeling and degeneration[3, 4]. In the early stage of the disease, anabolic mediators may correctly favour chondrocyte synthetic activity, while in advanced OA a panel of catabolic molecules including the most prominent inflammatory cytokines (e.g. TNF-α, IL-6 and IL-17A) participate in progressive cartilage matrix degradation and fibrotic remodeling[3, 4, 7, 8, 18, 41, 42]. Intriguingly, our data suggest that HA- and ESW-based treatment can decrease the inflammatory and to increase the anti-inflammatory cytokines production affecting OA patients. In fact, after HA and ESWT exposure, human primary cultured chondrocytes reduce the catabolic TNF-α, IL-6 and IL-17A, and enhance the regenerative IL-10 secretion[30, 31]. Our *in vitro* results are consistent with previous findings suggesting that such treatments may also attenuate the progression of cartilage degeneration, sustained by the production of various inflammatory molecules[8, 18, 43].

We propose that -through different and probably overlapping mechanisms- including the capacity to antagonize chondrocyte dedifferentiation, senescence, and production of inflammatory cytokines, as well as to enhance cell chondrogenic markers expression, proliferation, migration, and anabolic cytokine secretion, the aforementioned clinical strategies can support cartilage regeneration.

Interestingly, the possible correlation between inflammation, hyaluronan fragmentation, CD44 expression on chondrocytes, cartilage disruption and protective homeostatic tissue remodeling during OA[18, 41, 44–46] have already been reported although with contradictory results. In accordance with previous works[19, 20, 24], which revealed the synergistic properties of both low and high ranges of MW for *in vitro* treatment on human cultured chondrocytes, we used an innovative formulation of HA, consisting in a hybrid complex of combined low (LMW) and high molecular weight (HMW)-HA[24].

Previous reports suggest that, under arthritic diseases, several mediators -including enzymes and cytokines- actively contribute to increasing the amount of LMW-HA, which interferes in the HA-CD44 pathway[18, 47]. Therefore CD44 overexpression occurring during OA[18, 44–46] could represent -at early stages of inflammation- an attempt to increase protective cartilage regeneration through HA binding, which is limited by the fragmentation of large size HA[48].

Considering the central role of HA-CD44 interaction in tissue remodeling during OA progression[45], we then measured the chondrocyte expression of CD44 at the protein and molecular level.

We show for the first time that ESWT mediates CD44 overexpression on human OA cultured chondrocytes. Moreover, -after shockwave treatment- CD44 up-regulation on human cultured chondrocytes promotes a further HA-induced overexpression of the chondrogenic and anabolic process marker COL2A, as confirmed by the significant enhancement of the COL2A/COL1A ratio. Thus, ESW-mediated CD44 receptor increase on human chondrocytes may enhance their susceptibility to HA administration.

Taken together, these data display the presence of several molecules and mediators involved either in damage or in the healing of articular tissue, which can drive the cartilage regeneration, as well as SOX-9, COL1A-2A, p16, inflammatory cytokines and CD44 expression. Nevertheless, complete knowledge of the biological mechanisms triggered by ESWT, HA and PRP approaches may implement their use.

However, the main limitation of this work is its focus on primary cell cultures derived from OA explants, whereas a more exhaustive evaluation could also be performed on chondrocytes collected during the early stages of the disease. Enhancing susceptibility to HA administration through ESWT therefore represents an attractive therapeutic strategy against OA.

Further investigations should be conducted to better define the correct combination of strategies able to provide novel cues to improve *in vivo* articular cartilage repair, impairing a non-reparative fibrotic scarring response.

### Ethics approval and consent to participate

All research conducted was in compliance with the Declaration of Helsinki and was approved by the IRB (Institutional Review Board) of ‘‘Sapienza'' University and Sant’ Andrea Hospital (Rome, Italy). All participants gave informed written consent at the start of the study.

## Supporting information

S1 Fig Negative control for the immunofluorescence analysis of p16^INK4a^ expression. Negative control for the immunofluorescence analysis of p16^INK4a^ expression was achieved with GAM-FITC, in absence of anti-p16 immunolabeling (green). Nuclei are stained with DAPI. Photomicrographs are representative of one single culture.
(PDF)
