# Supplementary figures and images for: Hyaluronic Acid (HA), Platelet-Rich Plasm and Extracorporeal Shock Wave Therapy (ESWT) promote human chondrocyte regeneration in vitro and ESWT-mediated increase of CD44 expression enhances their susceptibility to HA treatment
Source: PLoS One. 2019 Jun 28;14(6):e0218740. doi: 10.1371/journal.pone.0218740 (PMC6599220; doi:10.1371/journal.pone.0218740)

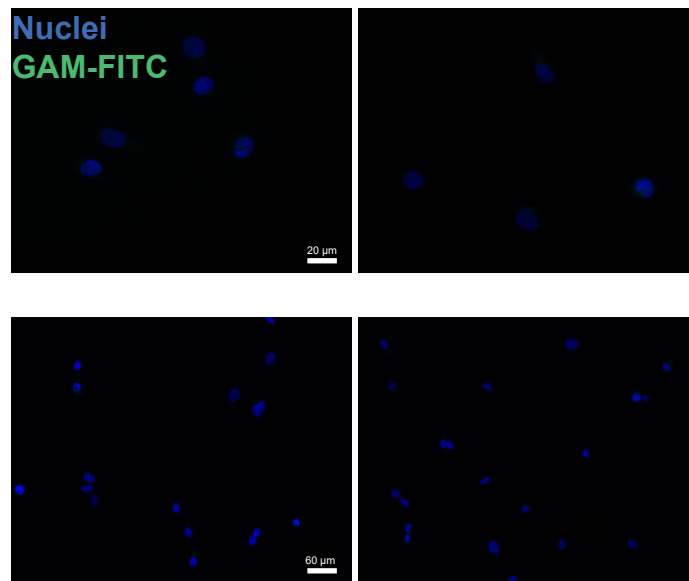

Supplementary Figure S1

Supplement: S1 Fig — Negative control for the immunofluorescence analysis of p16INK4a expression was achieved with GAM-FITC, in absence of anti-p16 immunolabeling (green). Nuclei are stained with DAPI. Photomicrographs are representative of one single culture. (PDF) [file pone.0218740.s001.pdf]
